# Cytotoxic Activity and Antibiofilm Efficacy of Biosynthesized Silver Nanoparticles against Methicillin-Resistant *Staphylococcus aureus* Strains Colonizing Cell Phones

**DOI:** 10.1155/2022/9410024

**Published:** 2022-03-25

**Authors:** Abderrahmen Merghni, Mohamed Ali Lassoued, Emira Noumi, Ramzi Hadj Lajimi, Mohd Adnan, Maha Mastouri, Mejdi Snoussi

**Affiliations:** ^1^Laboratory of Antimicrobial Resistance LR99ES09, Faculty of Medicine of Tunis, University of Tunis El Manar, Tunis, Tunisia; ^2^Laboratory of Pharmaceutical, Chemical and Pharmacological Drug Development LR12ES09, Faculty of Pharmacy, University of Monastir, Monastir, Tunisia; ^3^Department of Biology, College of Science, University of Hail, P.O. Box 2440, Ha'il, Saudi Arabia; ^4^Laboratory of Bioresources: Integrative Biology and Recovery, High Institute of Biotechnology, University of Monastir, Monastir 5000, Tunisia; ^5^Department of Chemistry, College of Science, University of Ha'il, P.O. Box 2440, Ha'il 81441, Saudi Arabia; ^6^Laboratory of Water Membranes and Environmental Biotechnologies, Center of Research and Water Technologies, P. B 273, 8020 Soliman, Tunisia; ^7^Laboratory of Transmissible Diseases and Biologically Active Substances LR99ES27, Faculty of Pharmacy, University of Monastir, Monastir, Tunisia; ^8^Laboratory of Genetics Biodiversity and Valorisation of Bioresources, High Institute of Biotechnology, University of Monastir, Monastir 5000, Tunisia

## Abstract

The interest for green synthesis of metallic nanoparticles (NPs) has acquired particular attention due to its low toxicity and economic feasibility compared with chemical or physical process. Here we carried out an extracellular synthesis approach of silver nanoparticles (AgNPs) using dried orange peel extract. Characterization studies revealed the synthesis of 25–30 nm AgNPs with distinct morphology as observed in transmission electron microscopes. Dynamic light scattering spectroscopy and Fourier transform infrared spectroscopy analyses further characterized nanoparticles confirming their stability and the presence of functional groups. The biological properties of biosynthesized AgNPs were subsequently investigated. Our results revealed anticancer activity of biogenic silver NPs against the B16 melanoma cell line with an IC50 value of 25 *µ*g/ml. Additionally, the developed AgNPs displayed a considerable antagonistic activity against methicillin-resistant *Staphylococcus aureus* (MRSA) strains colonizing cell phones, with inhibition zones between 12 and 14 mm and minimum inhibitory concentration values between 1.56 and 12.5 *µ*g/ml. Furthermore, the AgNPs exhibited potent antibiofilm activity against MRSA strains with the percent biofilm disruption reaching 80%. Our results highlighted the efficacy of biosynthesized AgNPs against bacterial biofilms and pointed to the exploration of orange peels as a natural and cost-effective strategy.

## 1. Introduction

Antimicrobial resistance is still one of the major problems threatening human health. It increases mortality and leads to longer hospital stays and greater medical costs [1]. Bacterial biofilm formation represents one of the most serious health hazards and leads to severe contamination problems. This microbial structure is difficult to combat since it has been revealed that antibiotic resistance is due to bacteria existing within a biofilm but rather to free bacteria [2]. The complexity of the bacterial biofilm structure mediated the inactivation or modification of antimicrobial enzymes and enabled the antimicrobial enzymes to penetrate the biofilm [3].

*Staphylococcus aureus* is one of the opportunistic pathogens often associated with serious nosocomial infections. Its virulence factors along with its multidrug resistance represent a major threat worldwide [4]. *S. aureus* is well known for its biofilm-forming ability involving various virulence factors such as bacterial surface proteins, fibronectin-binding proteins, the autolysin AtlA, protein A, biofilm-associated protein Bap, phenol-soluble modulins, proteases, nucleases, teichoic acids, the polysaccharide intercellular adhesin, and extracellular DNA [5]. Biofilm-forming capacity along with antibiotic resistance is crucial for the success of *S. aureus* as a dreaded pathogen in both healthcare and community settings. Moreover, methicillin-resistant *S. aureus* (MRSA) strains are usually resistant to commonly used antibiotics [6].

Nanotechnology is an emerging area of science dealing with nanoparticles of various materials.

Eco-friendly synthesis of silver nanoparticles can be used as a promising approach for multifunctional applications [7]. Hence, effective approaches for green synthesis of metallic NPs using natural resources such as plants, algae, bacteria, and fungi were largely reported [8]. Interestingly, plant extract-functionalized AgNPs have drawn considerable attention due to their exclusive biological properties, low toxicity, and good stability [9, 10]. The specific characteristics of nanoparticles such as their small size, various morphologies, and distribution improve their functional properties such as antioxidant, antiproliferative, antibacterial, and antibiofilm activities [7, 11].

The effectiveness of nanoparticles against drug-resistant bacteria as well as their virulence factors encourages further exploration in this field of research [12]. Silver nanoparticles obtained by green synthesis are largely investigated against various pathogenic microorganisms [13]. They are effectively used in the medical field as antimicrobial agents against active bacteria, viruses, and fungi [14]. The high solubility and the small size of nanoparticles represent specific characteristics to cross tissue barriers of drug-resistant bacteria. Such properties give this nanomaterial a great potential to reduce or limit the development of new mechanisms of resistance against them [15].

In this regard, we investigate the biosynthesis of AgNPs using dried orange peel extract, and subsequently, we evaluate its potentiality as antibacterial and antibiofilm agents against MRSA strains.

## 2. Materials and Methods

### 2.1. Preparation of the Extract

The dried peel of an orange (*Citrus sinensis*) was collected and cut into small pieces of approximately 1 cm. Peel pieces were then placed in a 250 mL conical flask containing 100 mL of double distilled water. After boiling for 30 min, the mixture was filtered to remove coarse pieces of peel and the sample was stored at 4°C before being used for the synthesis of AgNPs.

### 2.2. Biosynthesis of AgNPs

An aqueous solution of silver nitrate (AgNO_3_; Scharlab, Spain) was prepared and used for the synthesis of silver nanoparticles. Briefly, 10 mL of the prepared extract was added to 250 mL conical flasks containing 100 mL of AgNO_3_ (1 mM) and stirred continuously (200 tr/min) at 80 °C for 90 and 180 min. The color change of the mixture to brown indicates the formation of AgNPs. Once the process of biosynthesis was completed, nanoparticles were retained by ultracentrifugation at 14,000 rpm for 30 min, washed, and suspended in deionized water.

### 2.3. Characterization of Synthesized AgNPs

The reduction of silver ions into silver particles was monitored by UV-visible spectroscopy (Evolution 60, Thermo Scientific) at a wavelength of 300–800 nm. A dynamic light scattering spectroscopy (DLS) analysis was conducted to measure the particle size of the prepared AgNPs in an aqueous solution (hydrodynamic diameter) and to determine the polydispersity index (PDI) and zeta potential (surface charge) using a Zetasizer Nano-S (Malvern® instruments, UK) at 25°C. In order to avoid multiple scattering effects, the AgNPs were diluted 20- to 200-folds with deionized water. All measurements were undertaken in triplicate (*n* = 3) and results were expressed as mean ± SD. Fourier-transform infrared (FTIR) spectra obtained using an FTIR spectrophotometer (Perkin Elmer Spectrum FTIR Spectrometer, USA) at wavenumbers between 450 and 4000 cm^−1^ were conducted to determine the role of the phytoconstituents in NP synthesis. The AgNPs size was determined using transmission electron microscopy (TEM). High-resolution transmission electron microscopy (HR-TEM) was performed by using a TALOS F200x instrument (Thermo Fisher Scientific, Waltham, MA, USA). TEM analysis was performed at 200 kV and 5.5 *µ*A. ImageJ software (ImageJ 1.48v) was used to estimate the average particle size distribution.

### 2.4. Cytotoxic Activity of AgNPs

The cytotoxic effect of the prepared AgNPs was evaluated by the conventional MTT (3-[4, 5-dimethylthiazol-2-yl]-2, 5-diphenyltetrazolium bromide) reduction assay against B16 melanoma cell lines [16]. Cells were subcultured in DMEM (Sigma) supplemented with 10% FCS, 1% L-glutamine (200 mM), and 1% of mixture of penicillin (100 IU/ml) and streptomycin (100 IU/ml), at 37°C with 5% CO_2_. After distribution of 2.5 × 10^4^ B16 cells per well in 96-well plates, various concentrations of the AgNPs (viz., 3.125, 6.25, 12.5, 25, 50, and 100 *µ*g/ml) were added before the final incubation at 37°C for 24 h. A negative control with untreated cells was also evaluated. After treatment, the plates were incubated in the MTT solution (a final concentration of 0.5 mg/mL) for 3 h. The dark-blue formazan crystals that formed in intact cells were dissolved with DMSO, and the absorbance at 570 nm was measured with a spectrophotometer microplate reader (Bioteck, Elx 800). This colorimetric assay provides measurement of mitochondrial metabolic rate reflecting the viability in treated and untreated cells [17]. Results were expressed as the percentage of MTT reduction relative to the absorbance measured from negative control cells. All assays were performed in triplicate.

### 2.5. Antistaphylococcal Activities

#### 2.5.1. Microorganisms

Determination of the antibacterial and antibiofilm activities of biosynthesized AgNPs was carried out against the reference strain *S. aureus* ATCC 6538 and two MRSA isolates (68T and 12C) colonizing the mobile phones of Tunisian students. These strains were previously characterized for their biofilm formation ability [18].

#### 2.5.2. Disk Diffusion Method

Selected *S. aureus* strains were initially subcultured on nutrient agar medium. Then, the turbidity of an overnight-grown culture was adjusted to an optical density of 0.5 McFarland (McF). Each strain was spread over the Muller Hinton (MH) agar using sterile swabs. Disks of 6 mm diameter were placed on MH agar plate and impregnated with 50 *µ*g of AgNPs diluted in sterile 10% DMSO. The plates were then incubated at 37°C for 24 h and the appeared inhibition zones of the tested bacteria were measured. All assays were performed in triplicate [19].

### 2.6. Determination of Minimum Inhibitory and Minimum Bactericidal Concentrations

The minimum inhibitory concentration (MIC) values for AgNPs against MRSA strains were determined according to Merghni et al. [19]. The turbidity of an overnight grown culture was adjusted to 0.5 McF standards. The liquid dilution assay in MH broth was determined using a 96-well microtiter plate. The AgNPs were added aseptically to sterile 96-well microtiter plates (190 *μ*l per well) by twofold serial dilutions in Muller Hinton (MH) broth with 10% DMSO. The resultant doses of AgNPs in each solution (ranged between 50 *µ*g/ml and 0.09 *µ*g/ml) and the inocula (10 *μ*l) of each strain were added to each well. Wells containing only bacteria without AgNPs were considered as positive control. Negative control consists of wells made of sterile medium. Treated microplates were then incubated at 37°C for 24 h and the MIC was defined as the lowest concentration of the samples (AgNPs) that inhibited the bacterial growth.

To determine the minimum bactericidal concentration (MBC) values, a volume of 20 *μ*L from wells without bacterial visible growth was removed and plated on MH agar. Subsequently, the inoculated plates were incubated for 24 h at 37°C and the MBC was determined as the lowest concentration of the samples (AgNPs) that killed 99% of the tested bacteria [19].

### 2.7. Inhibition of Bacterial Cell Attachment by AgNPs

The antiadhesion properties of AgNPs against MRSA strains were tested following a microplate biofilm assay [20]. Overnight cultures grown in BHI broth were diluted to 10^6^ cfu/ml in BHI supplemented with 2% glucose (*w*/*v*). A 100 *μ*l aliquot for each strain was transferred to a 96-well microtiter plate, followed by the addition of 100 *μ*l of a subinhibitory concentration (1/16 to 1 × MIC) of the AgNPs dissolved in sterile BHI with 10% DMSO. The plates were kept undisturbed for 24 h at 37°C. Following incubation, the culture supernatant was discarded, and crystal violet (CV)-stained biofilm cells were determined at 570 nm using a microplate reader (D.E.E.D Reader, Bio-Rad Instruments). The percentage of inhibition of cell attachment was obtained by the following formula [20]:

(1) $$\begin{array}{c} \frac{\left(\text{OD growth control}-\text{OD sample}\right)}{\text{OD growth control}}\times 100. \end{array}$$

### 2.8. Reduction of Biofilm Growth and Development by AgNPs

Biofilms of *S. aureus* strains were allowed to develop for 48 h at 37°C in a 96-well microtiter plate, followed by the addition of the AgNPs. 100 *μ*l of the tested agent was dissolved in BHI supplemented with 10% DMSO to yield a range of concentrations of 1 × MIC, 2 × MIC, and 4 × MIC per well. The plates were further incubated for 24 h followed by an assessment of biofilm biomass by CV staining. CV-stained biofilm cells were quantified at 570 nm with the microplate reader, and the percentage of biofilm eradication was obtained by the following formula [20]:

(2) $$\begin{array}{c} \frac{\left(\text{OD growth control}-\text{OD sample}\right)}{\text{OD growth control}}\times 100. \end{array}$$

### 2.9. Statistical Analysis

All data were expressed as mean ± standard deviation (SD) from three independent experiments. Statistical analysis was performed with STATGRAPHICS Centurion XV, version 15.2.11 (StatPoint, Inc.). Differences between sample groups were analyzed using multiple-way analysis of variance (ANOVA) followed by Tukey's post hoc test. *p* value of <0.05 was considered significant.

## 3. Results and Discussion

### 3.1. Characterization of Biosynthesized AgNPs

**Figure 1:**
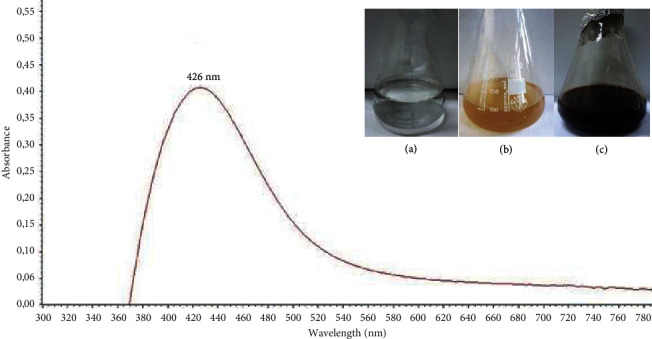
Ultraviolet-visible absorption spectra of AgNPs synthesis using the dried peel of orange (*Citrus sinensis*) extract (AgNO_3_ solution (a), aqueous orange peel extracts (b), and AgNPs (c)).

**Figure 2:**
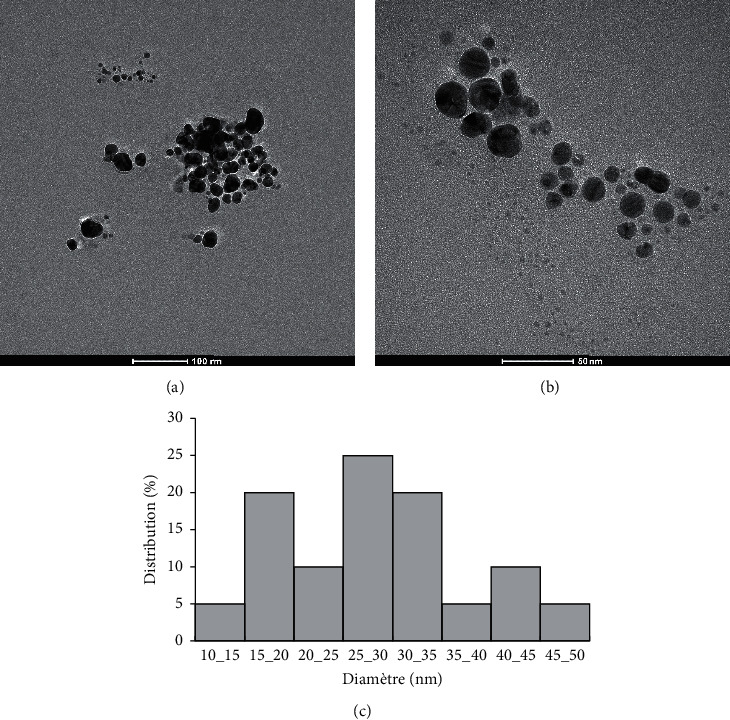
TEM analyses of biosynthesized AgNPs (a, b) using dried peel of orange (*Citrus sinensis*) extract and their size distribution (c).

**Figure 3:**
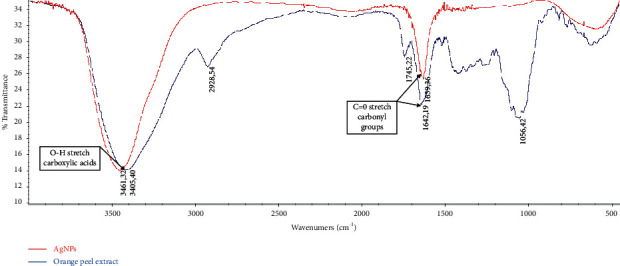
Fourier transform infrared spectroscopy (FTIR) analysis of biosynthesized silver nanoparticles (AgNPs) and the aqueous extracts of orange peel.

After addition of the dried orange peels extract to 1 mM of AgNO_3_, the color of the reaction mixture started changing to yellowish brown within 10 min. The final color (dark brown) of the solution deepened with an increase in time (Figure 1). One of the first indications of the biosynthesis of silver nanoparticles was represented by the color change of the reaction mixture as a visual marker [21]. The color transition reveals the biotransformation of the Ag + ion into Ag0 which indicates the biosynthesis of AgNPs [22, 23]. It was previously reported that the appearance of yellowish brown color in the reaction mixture of AgNPs solution was due to the excitation of surface plasmon vibrations [24]. Apart from the color change of the full reaction substrates, the formation of AgNPs using aqueous extracts from dried orange peels and AgNO_3_ (1 mM) solution was confirmed using ultraviolet-visible spectroscopy. Free electrons from metal nanoparticles such as silver and gold give rise to surface plasmon resonance (SPR) absorption band [25]. The characteristic SPR band of biogenic AgNPs occurs at 426 nm for reactions carried out at 80°C (Figure 1) indicating the reduction in the particle size at 80°C. The nucleation and growth mechanism affect nanoparticle formation. Particularly, smaller particle sizes were observed and a higher number of nuclei were formed at higher temperature [26]. The results of DLS of biosynthesized AgNPs using dried orange peel extract are shown in Table 1. The hydrodynamic diameter (nm) of biosynthesized AgNPs was 189.3 ± 30.19. Additionally, the zeta potential of the produced nanoparticles was −25.6 ± 0.08 (Table 1), indicating their stability. In fact, it was reported that a negative value of zeta potential indicates electrostatic repulsion among the particles, thereby increasing the stability of the formulation [27, 28]. The PDI was found to be 0.236 ± 0.04 (<0.3), reflecting a homogeneous and stable dispersion of the droplet size [29]. The transmission electron micrograph of the biosynthesized nanoparticles (Figure 2) showed the lack of agglomeration signs which confirmed their stabilization. The average size of these AgNPs was found to be 27.5 nm with a size range of 10 to 50 nm (Figure 2(c)). The particles with sizes 25–30 nm were the predominant, representing 25% of the total content. In general, the NPs were spherical, monodispersed, and uniformly distributed. Interestingly, monodispersity and stability are important and desired characteristics for commercial application of NPs [30]. The functional groups present in synthesized silver nanoparticles were determined by FTIR analysis (Figure 3). In the range of 3500 to 3400 cm^−1^, chemical groups corresponding to hydroxyl stretching (–OH) are noticeable in the structure. The double peaks at 1633 cm^−1^ point out the stretching vibration of the C=O group, suggesting the presence of carbonyl of nonsubstituted amide and water [31]. The existence of specific chemical groups in both solutions confirms the role of orange peel extract as a capping and reducing agent in the process of AgNPs biosynthesis. Thereby, the described phytochemical groups are implicated in the synthesis of nanoparticles through their interaction with metal salts [32].

### 3.2. Cytotoxic Activity of AgNPs

**Figure 4:**
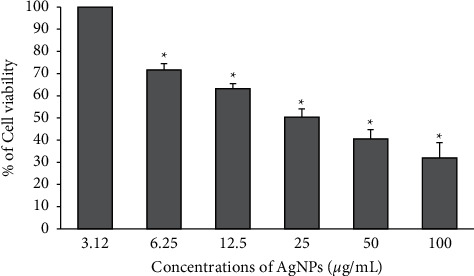
Percentages of viability of B16 melanoma cell lines treated with various concentrations of AgNPs after 24 h of exposure and evaluated by MTT reduction assay. Error bars represent standard deviations. ^*∗*^Differences were considered significant at *p* < 0.05.

The cytotoxic effect of the synthesized AgNPs was evaluated against the B16 melanoma cell line using the colorimetric assay (MTT).  Figure 4 shows the different percentages of viability of B16 cells exposed to various concentrations of AgNPs. Except for the concentration of 3.12 *µ*g/ml of AgNPs, other increased concentrations showed significant effects when compared to untreated cells (*p* < 0.05). The IC50 value were determined to be 25 *µ*g/ml. Another study reported the anticancer activity against the B16 melanoma cell line of biosynthesized gold nanoparticles using *Siberian ginseng* with an IC50 value of 10 *µ*g/ml [33]. The use of silver nanoparticles as a safe strategy for the development of anticancer therapy is of interest since they have remarkable effectiveness against skin cancer, wound care breast cancer, and cervical cancer [34–36]. Recently, biosynthesized metal NPs have attracted highlighted interest for their anticancer activity against a variety of tumor cells such as lung cancer cells [37], cervical cancer cells [38], and skin cancer cells [34]. Molecular mechanisms of metal NPs-induced cytotoxicity are related to the excessive production of reactive oxygen species (ROS) resulting in direct DNA damage and alteration in the mitochondrial membrane which consequently induces apoptosis and necrosis pathways [39, 40].

### 3.3. Antibiofilm Activity of AgNPs

**Figure 5:**
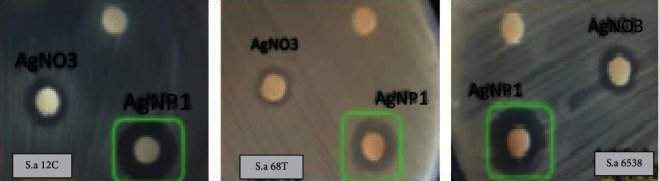
Antibacterial activity of silver nanoparticles (AgNP1) synthesized by reduction of silver nitrate (AgNO_3_) using dried peel orange extract. Shown is the inhibition zone of AgNO_3_ and AgNP1 against *S. aureus* 6538 (reference strain), *S. aureus* 12C, and *S. aureus* 68T (Tow MRSA colonizing cell phones).

Antistaphylococcal activities of AgNPs are reported as IZ, MIC, and MBC values in Table 2. MRSA strains 12C and 68T are sensitive towards the AgNPs with IZs of between 12 and 13.66 ± 0.58 mm (*p* < 0.05), respectively (Figure 5). Additionally, AgNPs displayed bacteriostatic effect against the majority of tested strains at a concentration of 12.5 *µ*g/ml. Interestingly, 1.56 *µ*g/ml of AgNPs are able to inhibit the bacterial growth of MRSA strain 12C. The MBC values of the tested agents were found to be similar to those against *S. aureus* strains (50 *µ*g/ml). The inhibition zones (IZs) found in our study are higher when compared to those reported by Kaviya et al. [26] showing IZs values between 7.8 and 9.2 nm against *S. aureus*. The AgNPs in their study were biosynthesized using fresh peels of *Citrus sinensis* unlike in our study which are dried peels. The superior activity of the AgNPs could be due to their small particle size (25–30 nm), facilitating easy passive transport through the cell membrane of treated bacteria. Our findings are in agreement with previous studies dealing with the biosynthesis of nanoparticles from silver nitrate using waste plant extracts and showing the enhancement of the antibacterial activity of green-synthesized AgNPs [13]. The mechanism through which AgNPs are active against bacteria lies mainly in their capacity to induce cell damage. Previously, the bactericidal effect of silver nanoparticles has been elucidated by various reports. Apart from their interaction with the bacterial cell surface, AgNPs can also cross the cell membrane, reach the cytoplasm [41], and attach to the DNA inhibiting its replication. It can also interact with the bacterial ribosome [42] or damage some enzymes structure, leading to eventual bacterial death [43]. Ample of studies have been performed to estimate the antibacterial potentials of AgNPs and revealed that these nanoparticles trigger oxidative stress, protein dysfunction, membrane and DNA damage, inducing microbial cell damage [44].

**Figure 6:**
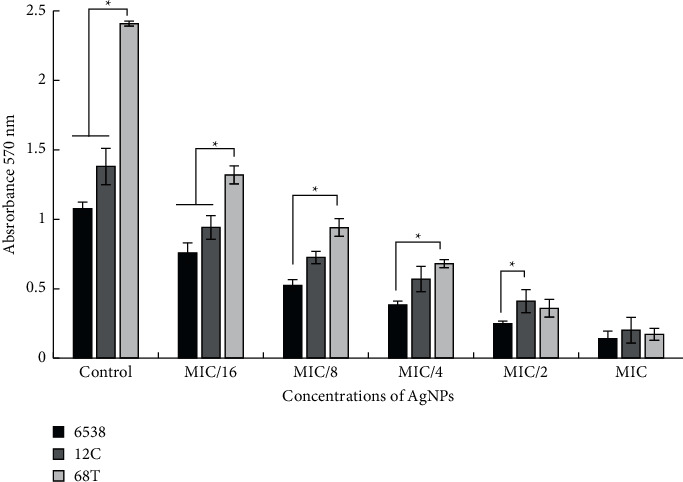
Evaluation of antiattachment effects of subinhibitory concentrations of biosynthesized AgNPs against *S. aureus* 6538, *S. aureus* 12C, and *S. aureus* 68T strains. Shown is the absorbance (A570) of stained bacteria with the crystal violet method. Error bars represent standard deviations. ^*∗*^Differences were considered significant at *p* < 0.05.

**Figure 7:**
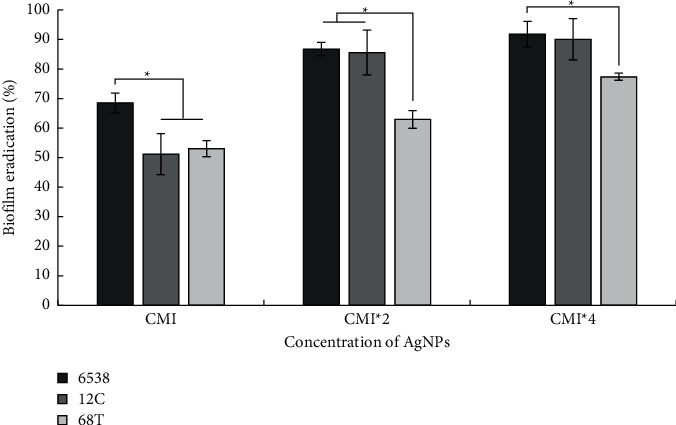
Effects of various concentrations (MIC; MIC^*∗*^2; MIC^*∗*^4) of dried peel orange AgNPs on the reduction of preformed biofilm of *S. aureus* strains (6538, 12C, 68T). Shown are the different percentages of biofilm eradication after staining with crystal violet. Error bars represent standard deviations. ^*∗*^Differences were considered significant at *p* < 0.05.

To evaluate the antiadhesion effects of AgNPs, the selected *S. aureus* strains were cultured in microtiter plates for 24 h in the presence of subinhibitory concentrations of the test agents (1/16× to 1×MIC). Our results showed that AgNPs are active against biofilm of all the tested strains even at a concentration of 1/8 × MIC, corresponding to 0,195 *µ*g/ml against the 68T strain and 1.56 *µ*g/ml against both 6538 and 12C strains (*p* < 0.05). With these concentrations, the absorbance at 570 nm was found to be less than 1 reflecting a weak biofilm capability of treated bacteria and subsequently an antiattachment effect which is a crucial phase (step) for biofilm formation and development [19]. AgNPs exerted an antiattachment effect, and they were more active against all tested *S*. *aureus* strains as shown in Figure 6. Here we present inhibition of bacterial adhesion at an early phase of biofilm formation. Generally, nanoparticles can interact with microbial biofilm in three steps. The first interaction concerns the transportation of these particles around the biofilm. The second one is their attachment to the surface of bacterial biofilm and finally the migration within this structure [45]. Biofilm formed by selected *S*. *aureus* strains were treated with biosynthesized AgNPs tested at various concentrations (CMI, 2 × CMI, and 4 × CMI). Our results showed various effects on the development of preformed biofilms with percent reduction values ranging from 51.18 ± 6.91 to 91.47 ± 4.36 (Figure 7). We noted that the AgNPs were more effective against the *S. aureus* ATCC 6538 strain known as biofilm-forming bacteria, with percent reduction values exceeding 91% at a concentration of 4 × MIC corresponding to 50 *µ*g/ml (*p* < 0.05). AgNPs were also highly effective against MRSA 12C strain with the percentage of biofilm eradication reaching 90.07 ± 6.91% (*p* < 0.05). Previously, the antibiofilm activity of AgNPs against Gram-positive and Gram-negative bacteria such as MRSA, *Streptococcus mutans*, *Acinetobacter baumannii,* and *Pseudomonas aeruginosa* as well as fungi *Candida albicans* was studied extensively and the potentiality of AgNPs was reported [46, 47]. Exposure to AgNPs leads to bacterial cell death due to stable adherence to the bacterial cell wall followed by penetration of the bacterium and rupturing of the cell membrane, reducing its permeability and respiration [41, 47].

## 4. Conclusion

The present study emphasized the biosynthesis, characterization, and assessment of cytotoxic and antibiofilm effects of silver nanoparticles. The obtained AgNPs from dried orange peel extract showed good stability and anticancer activity and exhibited strong antistaphylococcal and antibiofilm efficacy against tested strains. All the characteristics of these biosynthesized AgNPs incite their application as a potent antibacterial agent against MRSA biofilm as well as to prevent surface contamination and subsequent infections.

## Figures and Tables

**Table 1:** Characterization and stability of the synthesized AgNPs using orange peel extract.

Absorbance (nm)	Hydrodynamic diameter (nm)	Polydispersity index (PDI)	Zeta potentiel (mV)
426	189.3 ± 30.19	0.236 ± 0.04	−25.6 ± 0.08

**Table 2:** Antibacterial activity of biosynthesized AgNPs against methicillin-resistant *S. aureus* strains.

	AgNPs (mm ± SD)	Cefoxitin (30 *µ*g)	MIC (*µ*g/ml)	MBC (*µ*g/ml)
6538	14 ± 1	Sensitive	12.5	50
68T	13.66 ± 0.58	Resistant	1.56	50
12C	12 ± 0^*∗*^	Resistant	12.5	50

SD: standard deviation. ^*∗*^Significant difference (*p* < 0.05).

## Data Availability

All the original data, especially laboratory notebooks, are available on request.
